# Navigating the Statin Landscape: A Comprehensive Review of Stroke Prevention Strategies

**DOI:** 10.7759/cureus.53555

**Published:** 2024-02-04

**Authors:** Vinit Deolikar, Sarang S Raut, Saket Toshniwal, Sunil Kumar, Sourya Acharya

**Affiliations:** 1 Internal Medicine, Jawaharlal Nehru Medical College, Datta Meghe Institute of Higher Education & Research, Wardha, IND; 2 General Medicine, Jawaharlal Nehru Medical College, Datta Meghe Institute of Higher Education & Research, Wardha, IND; 3 Medicine, Jawaharlal Nehru Medical College, Datta Meghe Institute of Higher Education & Research, Wardha, IND

**Keywords:** preventive strategies, pleiotropic effects, cholesterol-lowering, cardiovascular diseases, stroke prevention, statins

## Abstract

This review provides a comprehensive exploration of the intricate relationship between statins and stroke prevention within the broader context of cardiovascular health. Delving into the mechanisms of statins, we elucidate their multifaceted contributions, ranging from cholesterol reduction to pleiotropic effects on the vascular system. Through a meticulous analysis of clinical trials, observational studies, and mechanistic investigations, we underscore the pivotal role of statins as integral components in the arsenal against strokes and associated cardiovascular events. The implications extend beyond statins as standalone interventions, emphasizing the potential for synergistic integration into broader stroke prevention strategies. Tailoring interventions to individual patient profiles and understanding the interplay with lifestyle modifications and other pharmacological approaches present opportunities for optimizing efficacy. Recommendations for future research advocate for continued exploration into the long-term effects of statin therapy, novel intervention combinations, and refined predictive models for personalized risk assessment. On a practical level, enhancing patient education, fostering interdisciplinary collaboration, and addressing barriers to medication adherence emerge as crucial aspects for real-world impact. In navigating this evolving landscape, the insights derived from this review contribute to informed decision-making and advancements in preventive cardiovascular medicine.

## Introduction and background

Cardiovascular diseases (CVDs) encompass a broad range of conditions affecting the heart and blood vessels, including coronary artery disease, heart failure, and stroke [1]. The intricate interplay of genetic, lifestyle, and environmental factors contributes to the development and progression of these diseases. As a leading cause of death, understanding the intricate mechanisms and risk factors associated with CVDs is paramount for devising effective preventive measures [1].

Among CVDs, strokes hold a distinctive position due to their acute and often irreversible consequences. Strokes, classified as ischemic or hemorrhagic, result from the disruption of blood flow to the brain, leading to neurological impairments [2]. The socio-economic burden of strokes, coupled with the emotional toll on affected individuals and their families, necessitates a concerted effort to explore and implement robust preventive strategies [3].

The pivotal role of statins in cardiovascular health has garnered significant attention in recent years. As lipid-lowering agents, statins not only address hypercholesterolemia but also exhibit potential pleiotropic effects that extend to the vascular system. In the landscape of stroke prevention, various strategies have been employed, ranging from lifestyle modifications to pharmacological interventions. This review seeks to critically examine the existing array of preventive measures, evaluating their effectiveness in diverse patient populations. By synthesizing current evidence, we aim to provide a comprehensive overview of the strategies that complement or synergize with statin therapy, offering insights into optimizing stroke prevention on both individual and population levels.

## Review

Statins: mechanism of action and overview

Introduction to Statins

Statins function by competitively blocking the active site of the key rate-limiting enzyme in the mevalonate pathway, known as HMG-CoA reductase. This inhibitory action prevents substrate access, impeding hydroxymethylglutaryl-CoA (HMG-CoA) conversion to mevalonic acid [4]. Through competitive inhibition of HMG-CoA reductase, the enzyme responsible for catalyzing the conversion of HMG-CoA to mevalonic acid, statins effectively curb endogenous cholesterol production [5]. The positive effects of statins primarily stem from their ability to reduce cholesterol biosynthesis, particularly in the liver, and their modulation of lipid metabolism achieved by inhibiting HMG-CoA reductase [6]. These inhibitors target the HMG-CoA reductase enzyme, resulting in a decrease in total low-density lipoprotein (LDL) and triglyceride concentrations, coupled with an increase in high-density lipoprotein (HDL) concentrations [7].

Mechanism of Action

Statins operate by inhibiting the HMG-CoA reductase enzyme, a critical enzyme in the mevalonate pathway, and the rate-limiting step in cholesterol biosynthesis. This inhibition effectively hinders HMG-CoA conversion to mevalonic acid, a precursor in cholesterol synthesis [4,5]. Through the reduction of cholesterol biosynthesis, statins bring about a decrease in LDL and triglyceride levels, concurrently elevating concentrations of HDL [6]. In addition to their lipid-lowering effects, statins exhibit pleiotropic effects contributing to their antiatherosclerotic properties. These include improvements in endothelial function, modulation of inflammatory responses, and maintenance of plaque stability [6,8]. These diverse effects beyond cholesterol regulation highlight the multifaceted impact of statins on cardiovascular health.

Commonly Prescribed Statins

Several commonly prescribed statins include atorvastatin (Lipitor), rosuvastatin (Crestor), simvastatin (Zocor), pravastatin (Pravachol), fluvastatin (Lescol), lovastatin (Altoprev), and pitavastatin (Livalo, Zypitamag) [7,9]. These medications manage and treat conditions such as hypercholesterolemia, atherosclerosis, and other disorders associated with elevated cholesterol levels [7,9]. The mechanism of action for statins involves the inhibition of the HMG-CoA reductase enzyme, which serves as the rate-limiting enzyme in the mevalonate pathway [4,5]. By impeding cholesterol biosynthesis, statins effectively reduce levels of LDL and triglycerides while simultaneously increasing concentrations of HDL [7]. It is crucial to recognize that statins may not be suitable for everyone, and individuals should consult with their healthcare providers to determine the most appropriate statin and dosage for their specific circumstances [9]. This personalized approach ensures that the benefits of statin therapy are maximized while minimizing potential risks and addressing individual health considerations.

Safety and Side Effects

Statins are generally considered safe and well-tolerated; however, like any medication, they may elicit side effects in some individuals. The most commonly reported side effect of statins is myalgia, or muscle pain, occurring in 1-10% of patients [10]. While relatively rare, rhabdomyolysis is a serious side effect that can lead to muscle breakdown and kidney damage, affecting less than 0.1% of patients [10]. Additionally, potential side effects of statins encompass liver enzyme abnormalities, gastrointestinal symptoms, and neurological issues such as memory loss, forgetfulness, and confusion [8,9]. Certain statins, particularly those metabolized by CYP 3A4, may interact with other drugs, including clarithromycin, protease inhibitors, cyclosporine, gemfibrozil, and oral contraceptives [8]. Individuals are advised to engage in thorough discussions with their healthcare providers before commencing statin therapy to address any potential side effects or concerns. This proactive approach ensures that the benefits of statin treatment are weighed against potential risks, and appropriate measures can be taken to mitigate adverse effects in a personalized manner.

Statins and CVDs

Role of Statins in CVD Prevention

Statins have proven efficacy in preventing CVD events and reducing all-cause mortality in adults aged 40 to 75 years who exhibit one or more CVD risk factors, such as dyslipidemia, diabetes, hypertension, or smoking [11]. Additionally, statins have demonstrated effectiveness in reducing the risk of a first CVD event in otherwise healthy individuals at high risk [12]. To guide clinical practice, the United States Preventive Services Task Force (USPSTF) recommends statin prescription for the primary prevention of CVDs in adults aged 40 to 75 years with one or more CVD risk factors and a calculated 10-year CVD event risk of 10% or greater [11]. The mechanism of action underlying statin effectiveness involves the reduction of cholesterol biosynthesis, primarily in the liver, achieved through inhibiting HMG-CoA reductase [13]. Beyond lipid regulation, statins exhibit pleiotropic effects, including improvements in endothelial function, modulation of inflammatory responses, and maintenance of plaque stability, all contributing to their antiatherosclerotic effects [11,12]. Statins emerge as a safe and effective option for the primary prevention of CVDs in high-risk individuals. For personalized recommendations, individuals are encouraged to engage in discussions with their healthcare providers to determine the most suitable statin and dosage tailored to their specific circumstances [11].

Evidence Supporting Statin Use in Reducing Cardiovascular Events

Ample evidence supports the efficacy of statins in mitigating cardiovascular events. These medications demonstrate effectiveness in averting CVD events and reducing overall mortality among adults aged 40 to 75 who possess one or more CVD risk factors, such as dyslipidemia, diabetes, hypertension, or smoking. Pooled analyses of all 12 trials indicate that statin therapy is associated with a modest reduction in cardiovascular mortality risk over a two to six-year period [11]. Furthermore, the data reveals that statin therapy is linked to a decreased risk of composite cardiovascular outcomes, as evidenced by a relative risk (RR) of 0.72 (95% CI, 0.64 to 0.81) and an absolute risk difference (ARD) of -1.28% [11]. A pooled analysis of 15 trials consistently supports these findings, reinforcing the positive impact of statin therapy on composite cardiovascular outcomes [11]. Among the 12 trials reporting on cardiovascular mortality (encompassing 75,138 participants), only the WOSCOPS trial (West of Scotland Coronary Prevention Study; n = 6595) revealed a statistically significant difference between statin and placebo in the risk of cardiovascular mortality at six years (ARD, -0.70% (95% CI, -1.36% to -0.05%)) [11]. These collective findings underscore the crucial role of statins in the primary prevention of CVDs in high-risk individuals. However, individuals must discuss with their healthcare providers to ascertain the most appropriate statin and dosage tailored to their specific circumstances [11]. This personalized approach ensures informed decision-making and optimal cardiovascular risk management.

Statins and Atherosclerosis

Statins have been identified as playing a pivotal role in preventing and treating atherosclerosis, a condition characterized by the accumulation of plaque in the arteries, ultimately contributing to CVDs [14]. Studies have revealed that statins contribute to the reduction of lipid content within atherosclerotic plaques, facilitate the stabilization of plaques with thickened fibrous caps, and promote macrocalcification. This process enhances the stability of atheromas [14]. Notably, a study demonstrated that statins can disrupt macrophage Rac1 regulation, resulting in increased calcification of atherosclerotic plaques [15]. The potential benefits of statins in atherosclerosis prevention and treatment were underscored by a case demonstrating significant regression of coronary plaques with statin therapy [14]. These findings suggest that statins may offer a multifaceted role in addressing atherosclerosis beyond their well-known cholesterol-lowering effects. Nevertheless, individuals are strongly advised to engage in consultations with their healthcare providers to determine the most suitable statin and dosage tailored to their specific circumstances [16]. This personalized approach ensures that the potential benefits of statin therapy in preventing and treating atherosclerosis are maximized while considering individual health considerations.

Statins and stroke prevention

Overview of Statins in Stroke Prevention

The utilization of statins for stroke prevention remains a subject of ongoing research and debate within the medical community. Divergent findings from various studies contribute to the uncertainty surrounding the efficacy of statins in reducing the risk of stroke. A meta-analysis of randomized controlled trials did indicate a reduction in the overall incidence of stroke with statin use, although the statistical significance of this reduction was relatively low. Additionally, the analysis suggested a potential increase in the incidence of fatal stroke, although this effect did not reach statistical significance. The conflicting outcomes of large-scale clinical trials further contribute to the uncertainty, with some trials highlighting the beneficial effects of statins in stroke prevention while others do not [17]. Despite this debate, there is strong evidence suggesting that statins are associated with a reduction in the absolute risk of ischemic strokes and cardiovascular events [18]. However, the overall consensus and guidelines regarding the use of statins for stroke prevention may vary. Individuals are strongly advised to consult with their healthcare providers to determine the most appropriate stroke prevention strategies tailored to their circumstances. This personalized approach ensures that decisions regarding statin use consider individual health factors and align with the latest evidence and guidelines.

Mechanisms Through Which Statins May Prevent Stroke

The potential mechanisms through which statins may prevent strokes involve many physiological processes. One significant avenue is their antioxidative effects, where statins inhibit Rac1 and NADPH oxidase, protecting the body from oxidative stress and thereby reducing the risk of atherosclerosis [19]. Another critical aspect is the stabilization of atherosclerotic plaques, which statins achieve by influencing macrophage cholesterol metabolism and activation, ultimately promoting plaque stability and preventing rupture-a crucial factor in the occurrence of strokes [20]. Additionally, a prevalent hypothesis suggests a direct pleiotropic effect of statin therapy on atherosclerotic plaques, further contributing to their stability [18]. In addition to plaque stabilization, statins benefit by reducing lipid and platelet aggregation, lowering the risk of CVDs, and indirectly contributing to stroke prevention [17]. Their capacity to enhance endothelial function underscores their role in mitigating the risk of strokes [17]. Furthermore, statins display anti-inflammatory activity, a vital attribute in decreasing the overall risk of CVDs and, by extension, strokes [17]. Some studies even propose a direct neuroprotective action of statins, indicating a potential for preventing strokes or reducing their severity [17]. However, the efficacy of statins in stroke prevention remains a subject of ongoing research and debate. Discrepancies in large-scale clinical trials have led to uncertainty regarding their overall effectiveness in this specific domain [17]. Despite promising findings, the complex interplay of various factors necessitates continued investigation to establish a more comprehensive understanding of statins' role in preventing strokes.

Clinical Trials and Studies on Statins and Stroke Prevention

The role of statins in stroke prevention has been extensively examined through various studies and meta-analyses, resulting in diverse findings that contribute to ongoing research and debate in this area. One meta-analysis of randomized controlled trials demonstrated a reduction in the overall incidence of stroke with statin use, although the statistical significance of this reduction was modest. Additionally, the analysis suggested a potential increase in the incidence of fatal stroke, but this effect did not reach statistical significance [17]. On the other hand, evidence strongly indicates that statins are associated with a reduction in the absolute risk of ischemic strokes and cardiovascular events [18]. However, a different meta-analysis presented a contrasting perspective, suggesting that statins may not have a definite preventive effect on strokes [21]. The effectiveness of statins in stroke prevention remains a subject of ongoing research and debate, with studies yielding disparate results. While some studies demonstrate beneficial effects, others do not, contributing to uncertainty regarding the overall efficacy of statins in preventing strokes [13,17,18]. The complexities surrounding these findings underscore the need for continued research to gain a more comprehensive understanding of the nuanced effects of statins on stroke prevention.

Effectiveness of statins in different patient populations

Primary Prevention in High-Risk Populations

A cohort study on a population basis discovered that statins effectively reduced cardiovascular events among adherent individuals across 10-year coronary risk groups. The number needed to treat (NNT) in the lower-risk categories (<5% and 5-7.4%) was higher compared to the moderate and high-risk categories (7.5-9.9% and 10-19.9%) [22]. According to a meta-analysis of randomized trials, the use of statins in individuals with low cardiovascular risk resulted in a 10% reduction in the relative risk of death from any cause [23]. The USPSTF recommends that healthcare providers initiate statins for the primary prevention of CVDs in adults aged 40 to 75 years who exhibit one or more CVD risk factors and have a calculated 10-year CVD event risk of 10% or greater [11]. However, the efficacy of statins for primary prevention in older adults is constrained, and the benefit of statins in preventing atherosclerotic CVD (ASCVD) events in this demographic remains uncertain [24].

Secondary Prevention in Patients With a History of Stroke

Numerous studies and meta-analyses have endeavored to assess the efficacy of statins in secondary stroke prevention. A meta-analysis of randomized controlled trials revealed that statins led to a 20% reduction in the overall incidence of stroke, with an RR reduction of 21% and an absolute risk reduction of 0.9% [18]. Another comprehensive meta-analysis, incorporating both randomized controlled trials and observational cohort studies, indicated that statins diminished the odds of stroke of any type and recurrence of ischemic stroke [25]. However, a more recent meta-analysis suggested that statins may not definitively prevent strokes [21]. The evidence suggests that statins might be effective in reducing the risk of stroke in individuals with a history of stroke. Nevertheless, the efficacy appears to vary depending on the specific study and population under consideration. Individuals are strongly advised to engage in discussions with their healthcare providers to determine the most appropriate stroke prevention strategies for their circumstances. This personalized approach ensures that decisions about statin use and other preventive measures are tailored to each individual's unique needs and health considerations.

Age and Gender Considerations

The effectiveness of statins across different age groups has been a subject of research. According to a review by the USPSTF, the benefits of statins appear similar in patient groups defined by demographic characteristics, such as sex and race, as well as clinical characteristics, such as the presence of diabetes or kidney dysfunction. However, evidence on how statin benefits vary by age is limited for older individuals, as most trials have participants with a mean age in their 50s and 60s. The review emphasized that statin benefits are not restricted to a specific age group, with similar relative risk estimates observed in subgroups stratified according to baseline age. Moreover, the absolute benefits of statin therapy were proportionately greater in patients at higher baseline risk [16]. A meta-analysis of individual participant data from 28 randomized controlled trials published in The Lancet concluded that statin therapy significantly reduces major vascular events regardless of age [26]. Additionally, a population-based cohort study found that statins effectively reduced cardiovascular events across 10-year coronary risk groups in adherent individuals, with the NNT being higher in the lower-risk categories than the moderate and high-risk categories [22]. Although evidence on the benefits of statins in older individuals remains limited, the available data suggests that statins effectively reduce cardiovascular events across various age groups. Further research may be necessary to provide more comprehensive insights into the effectiveness of statins in specific age populations.

Statin Use in Special Populations

Statins demonstrate effectiveness in reducing cardiovascular events among patients with diabetes. A meta-analysis of randomized controlled trials revealed that statins led to a 21% reduction in the incidence of major cardiovascular events in individuals with diabetes [27]. Another meta-analysis focusing on randomized controlled trials found that statins decreased the risk of major vascular events in patients with diabetes by 21% [28]. In alignment with these findings, the American Diabetes Association recommends moderate-intensity statin therapy for all patients aged 40 to 75 with diabetes, irrespective of baseline lipid levels [29]. Furthermore, statins have proven effective in reducing cardiovascular events in patients with chronic kidney disease (CKD). A meta-analysis of randomized controlled trials indicated that statins reduced the incidence of major cardiovascular events in individuals with CKD by 20% [11]. The American College of Cardiology/American Heart Association guidelines endorse moderate-intensity statin therapy for patients with CKD, regardless of baseline lipid levels [27]. The accumulated evidence strongly suggests that statins are effective in reducing cardiovascular events in patients with both diabetes and CKD. These findings underscore the significance of statin therapy in mitigating cardiovascular risks in these specific patient populations.

Combination therapies and statin combinations

Combination Therapies for Enhanced Stroke Prevention

Numerous studies and meta-analyses have consistently demonstrated the effectiveness of statins in the prevention of strokes. A meta-analysis of randomized controlled trials found that statins led to a 20% reduction in the overall incidence of strokes, with an RR reduction of 21% and an absolute risk reduction of 0.9% [29]. Robust evidence also strongly indicates that statins are associated with a reduced absolute risk of ischemic strokes and cardiovascular events [18]. In line with these findings, guidelines from the American Heart Association (AHA) and the American Stroke Association (ASA) emphasize the utility of statin therapy for secondary prevention of strokes, particularly in patients with a history of ischemic stroke [25]. Moreover, statins are now recommended for the primary prevention of ischemic strokes in patients assessed to have a high 10-year risk for cardiovascular events [30]. These collective findings provide substantial support for the use of statins as an effective strategy for stroke prevention, both in primary and secondary prevention scenarios.

Interaction With Other Medications

The interaction between statins and other medications is a critical consideration in healthcare. While statins are generally well-tolerated, their potential interactions with other drugs can result in adverse effects or diminished efficacy. Certain antibiotics, antifungals, and antivirals may interact with statins, heightening the risk of muscle-related side effects. Additionally, drugs used for heart conditions and high blood pressure can affect the metabolism of statins, potentially increasing the likelihood of side effects. Therefore, healthcare providers must meticulously review patients' medication lists when prescribing statins to identify and manage potential interactions. Patients also play a crucial role in ensuring their safety. Individuals must inform their healthcare providers about all medications, including over-the-counter drugs and supplements, to minimize the risk of harmful interactions. Patient education is key, with individuals being made aware of the signs and symptoms of potential side effects. Importantly, patients should be advised to seek medical attention promptly if they experience any concerning symptoms while taking statins in combination with other medications. This proactive approach, involving both healthcare providers and patients, is essential to ensure statins' safe and effective use in conjunction with other therapeutic interventions.

Statin Combinations and Their Effectiveness

The exploration of combination therapies, including the use of statins in conjunction with other medications, is a crucial aspect of stroke prevention and treatment. Studies have delved into the combination of statins with other medications, such as antihypertensives, for primary stroke prevention. Notably, the HOPE-3 trial (Heart Outcomes Prevention Evaluation-3) demonstrated that combining antihypertensive therapy with a statin reduced the incidence of first strokes among individuals at intermediate cardiovascular risk [31]. Statin therapy is commonly employed in combination with other medications for lipid management. Various intensities of statin therapy, including high-intensity, moderate-intensity, and low-intensity, lead to different reductions in LDL-C levels: ≥50 percent, 30 to 49 percent, and <30 percent, respectively [32]. The AHA/ASA Guidelines recommend statin therapy for primary stroke prevention in specific cases, with supporting studies aligning with these guidelines [25]. However, there are limitations to these guidelines, as they mainly demonstrate the benefits of statins in preventing recurrent stroke without providing conclusive evidence for other outcomes, such as ischemic or hemorrhagic stroke [25]. A study examining the efficacy of statins in preventing hemorrhagic stroke revealed a higher incidence of hemorrhagic stroke, particularly in older patients treated with statins [17]. While combination therapies and statin combinations with other medications can play a role in stroke prevention and treatment, healthcare providers must carefully evaluate each patient's unique circumstances. Balancing these therapies' potential benefits and risks is essential to ensure an informed and tailored approach to stroke prevention and treatment.

Adherence and patient education

Importance of Patient Adherence to Statin Therapy

Adherence to statin therapy plays a crucial role in stroke prevention and treatment. Studies indicate that patient-reported medication-taking behaviors are associated with statin PDC (proportion of days covered), with those perceiving lower cardiovascular risk being less likely to adhere [33]. Various approaches to enhance statin adherence, including patient education, support, reminders, and efforts to simplify regimens and reduce costs, have shown positive but relatively modest effects on adherence [33]. Tailoring interventions to individual attitudes and actual or perceived CVD risk could enhance their effectiveness [33]. Several factors contribute to non-adherence to statin therapy, including age, sex, race, socioeconomic status, side effects, and comorbidities [34]. Patients may report adverse effects, such as muscle symptoms, leading to medication intolerance [34]. To improve adherence to statin therapy, patients may benefit from extended discussion time with their clinician, written information about statin risks, side effects, and drug interactions, as well as reminders through mail and telephone communication [35]. Audio booklets have also proven effective in increasing patients' knowledge and understanding of statin medication, potentially enhancing adherence [35]. Healthcare providers should actively elicit and educate patients about their adherence behaviors and ASCVD risks, tailoring interventions to individual attitudes and perceived CVD risks to improve adherence. Providing additional time for discussion, written information, and reminders through various channels can contribute to better adherence to statin therapy, thereby enhancing stroke prevention and treatment outcomes.

Strategies for Improving Patient Education

Patient education plays a pivotal role in enhancing adherence to statin therapy, and employing various strategies can significantly contribute to improved patient understanding and compliance with statin regimens. Firstly, clinicians must assess a patient's health literacy and knowledge before implementing educational techniques. This ensures that the education strategies chosen are tailored to the individual patient, considering their needs and comprehension level [36]. One effective method is utilizing the patient teach-back technique, where patients are asked to articulate, in their own words, the information related to their health. This approach helps confirm that the patient has grasped the necessary information and can apply it effectively [36].

Tailoring educational materials to the patient's preferences is another valuable strategy. Offering educational materials in formats preferred by the patient, such as audio booklets or visual aids, has enhanced understanding and adherence [37]. Similarly, incorporating educational technology, such as interactive online resources or educational videos, can complement traditional methods and improve patient education [37]. Recognizing and accommodating the patient's learning style is crucial. Whether the patient is a visual, auditory, or kinesthetic learner, understanding their preferred learning method allows healthcare providers to tailor their education approach accordingly [38]. Moreover, effective communication is paramount. Clear and simple language should be used, avoiding medical jargon that may hinder patient comprehension. This approach fosters a better understanding of statin therapy and promotes adherence to treatment regimens [38]. By implementing these diverse strategies, healthcare providers can enhance patient education, facilitate a better understanding of statin therapy, and ultimately promote improved adherence to treatment regimens. This is particularly vital in the context of stroke prevention and management, where adherence to statin therapy plays a crucial role in achieving optimal outcomes.

Addressing Patient Concerns and Misconceptions

Addressing patient concerns and misconceptions is crucial for enhancing adherence to statin therapy. Healthcare providers play a pivotal role in this process by employing various communication strategies. Open communication is fundamental, and healthcare providers should actively encourage patients to express their worries, fears, and any potential misconceptions about statin therapy [39]. Establishing an open dialogue fosters a better understanding of the patient's perspective.

A patient-centered approach is essential to tailor the intervention to individual patient needs. By utilizing open-ended questions, actively listening, and customizing education based on the patient's unique preferences, healthcare providers can address concerns in a personalized manner [36]. Empathetic communication is a key element in addressing patient concerns. Healthcare providers should approach discussions with empathy and understanding to build trust and enhance the patient's receptiveness to information about statin therapy [40].

Clear and simple language is crucial during discussions to avoid medical jargon that could lead to misunderstandings. By employing easily understandable language, healthcare providers can communicate effectively and improve patient comprehension of statin-related information [39]. Providing written information can be a valuable supplement to verbal communication. Offering written materials that outline statin risks, side effects, and potential drug interactions can help clarify patient concerns and improve their overall understanding, ultimately contributing to better adherence to therapy [35]. By implementing these comprehensive strategies, healthcare providers can effectively address patient concerns and misconceptions, leading to improved adherence to statin therapy. This, in turn, enhances the overall effectiveness of stroke prevention and treatment.

## Conclusions

In conclusion, this comprehensive review has illuminated the multifaceted role of statins in stroke prevention, emphasizing their significance as pivotal agents in reducing the risk of cardiovascular events. The synthesis of evidence from clinical trials, observational studies, and mechanistic investigations underscores the diverse contributions of statins, from cholesterol reduction to pleiotropic effects on the vascular system. Moving beyond the standalone efficacy of statins, the implications drawn from our findings underscore the potential for synergistic integration within broader stroke prevention strategies. Tailoring interventions to individual patient profiles and understanding the interplay with lifestyle modifications and other pharmacological approaches present avenues for optimizing the efficacy of preventive measures. Looking ahead, our recommendations advocate for continued research into the long-term effects of statin therapy, exploring novel intervention combinations, and refining predictive models for personalized risk assessment. On the practical front, improved patient education, interdisciplinary collaboration, and efforts to address barriers to medication adherence are essential for enhancing the real-world impact of statins on stroke prevention. As we navigate this evolving landscape, the insights derived from this review contribute to the ongoing discourse shaping strategies for mitigating the global burden of strokes and advancing cardiovascular health.
